# Ten-Year Research Update Review: Antiviral Activities from Marine Organisms

**DOI:** 10.3390/biom10071007

**Published:** 2020-07-07

**Authors:** Gennaro Riccio, Nadia Ruocco, Mirko Mutalipassi, Maria Costantini, Valerio Zupo, Daniela Coppola, Donatella de Pascale, Chiara Lauritano

**Affiliations:** 1 Marine Biotechnology Department, Stazione Zoologica Anton Dohrn, CAP, 80121 Naples, Italy; gennaro.riccio@szn.it (G.R.); nadia.ruocco@szn.it (N.R.); mirko.mutalipassi@szn.it (M.M.); maria.costantini@szn.it (M.C.); valerio.zupo@szn.it (V.Z.); daniela.coppola@szn.it (D.C.); donatella.depascale@szn.it (D.d.P.); 2 Institute of Biosciences and BioResources (IBBR), National Research Council, Via Pietro Castellino 111, 80131 Naples, Italy; 3 Institute of Biochemistry and Cell Biology (IBBC), National Research Council, Via Pietro Castellino 111, 80131 Naples, Italy

**Keywords:** marine organisms, antiviral, marine natural products, viruses

## Abstract

Oceans cover more than 70 percent of the surface of our planet and are characterized by huge taxonomic and chemical diversity of marine organisms. Several studies have shown that marine organisms produce a variety of compounds, derived from primary or secondary metabolism, which may have antiviral activities. In particular, certain marine metabolites are active towards a plethora of viruses. Multiple mechanisms of action have been found, as well as different targets. This review gives an overview of the marine-derived compounds discovered in the last 10 years. Even if marine organisms produce a wide variety of different compounds, there is only one compound available on the market, Ara-A, and only another one is in phase I clinical trials, named Griffithsin. The recent pandemic emergency caused by SARS-CoV-2, also known as COVID-19, highlights the need to further invest in this field, in order to shed light on marine compound potentiality and discover new drugs from the sea.

## 1. Introduction

Oceans cover more than 70% of the surface of our planet [1]. The vast ocean extension and its unique environments are characterized by huge taxonomic and chemical diversity of marine organisms [2,3], and it has been classified, already in the 1980s, as the largest reservoir of natural products to be evaluated for their activity as drugs [4]. Recently, several projects worldwide, such as those funded by European Union under the FP7 and H2020 frameworks, focused on the exploitation of marine organisms in order to identify new products for applications in different industrial sectors (e.g., pharmaceutical, nutraceutical, cosmeceutical, aquaculture, and energy sectors) [5]. In addition, many of these projects, under the topic “Blue growth”, focused on more environmental-friendly approaches to drug discovery in order to identify new lead compounds for the treatment of human pathologies without any negative impact on the marine environment and focusing on easily cultivable organisms, especially microorganisms.

It is well documented that humans, animals, plants, fungi, and bacteria produce metabolites to protect themselves against various pathogens. Viruses are the most abundant entities of the Ocean and, although their existence has been known for many years, they have recently been recognized as important factors influencing microbial communities [6], causing marine organism mortality, and driving global geochemical cycles [7]. In addition, various studies investigated if they can be responsible for the decline of particular species, such as populations of sockeye (*Oncorhynchus nerka*) and Chinook (*Oncorhynchus tshawytscha*) salmon in the Northeast Pacific [8]. Previously unknown viruses have been found in dead and dying farmed salmon, highlighting their potential role in population dynamics of wild fish stocks, and the threat they may pose to aquaculture [8]. Marine organisms are known to produce antiviral compounds that can have pharmaceutical applications [9]. Despite the huge biological and chemical biodiversity of marine organisms, only one marine derived compound with antiviral activity reached the market until now, Vidarabine (Ara-A). Ara-A (a nucleoside extracted from a sponge), Food and Drug Administration (FDA) approved in 1976, is actually used as antiviral drug against Herpes Simplex Virus (HSV) (https://www.midwestern.edu/departments/marinepharmacology/clinical-pipeline.xml).

In addition, actually there are only 12 marine compound-derived drugs available on the market, and about 24 natural products in Phase I to Phase III clinical trials [2]. One compound out of 24, named Griffithsin (a lectin extracted from a red algae), suggested for anti-HIV activity, is in clinical trials. Viruses are known to produce damages to marine organisms, as well as to terrestrial animals and plants, and causing damages to humans and economies.

Even if there have been several studies on understanding viral physiology and suitable treatments and vaccines over the past half of century, still several infections, such as those due to Human Immunodeficiency Virus (HIV), Hepatitis C Virus (HCV), and more recently coronavirus, affect a substantial proportion of the world populations of different ages, causing thousands of deaths annually. There are no definite vaccines against numerous viral infections (e.g., for the Measles virus [10]), and further research is necessary to find effective antivirals as alternative therapies that could contribute to outbreak containment and lead to eradication. In addition, the development of viral resistance to antiviral drugs and side effects like toxicity have continuously stimulated the search of new antiviral compounds. Common is the search for compounds with distinct/specific mechanisms of action, good bioavailability and very low toxicity. Mechanisms of action of possible antiviral compounds are various because they can block viruses at different stages of their life cycles (Common viral life cycle stages are attachment, penetration, uncoating, replication, assembly, and release) [11]. However, the comparison of marine natural product activity is sometimes difficult, because there are several methods used to assess antiviral capacity (such as cell viability, syncytia formation, viral titration by Real Time-qPCR and virus plaque reduction assay). The aim of this review is to summarize the last 10-year research on antiviral compounds isolated from marine organisms, from bacteria to vertebrates.

## 2. Marine Bacteria and Fungi

Bacteria and fungi are widely distributed in marine environments (from shallow water to deep sea, even down to the polar ice covers), and synthesize a high number of structurally and functionally diverse bioactive molecules. Although these compounds have been shown to have several bioactivities, to date there are limited studies of microbial natural products with antiviral activity, especially in the last ten years (Table 1 and Table 2). 

### 2.1. Marine Bacteria

**Figure 1:**
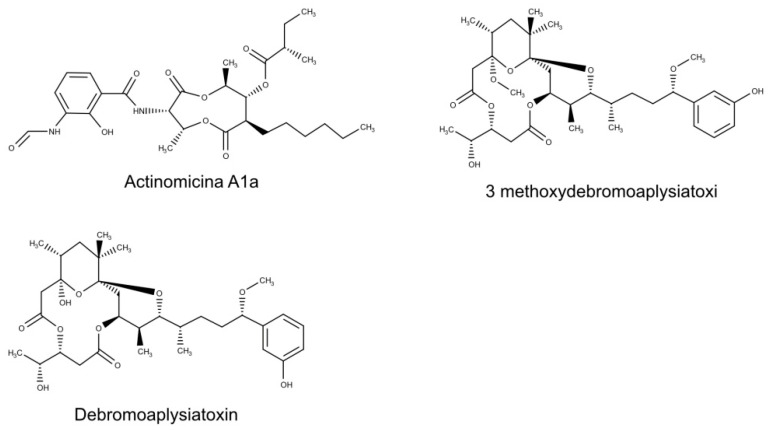
Structures of compounds isolated from marine bacteria with antiviral activity.

Actinobacteria, especially from the genus *Streptomyces*, represent a rich source of biologically active molecules [33,34,35,36,37,38,39]. Jakubiec-Krzesniak and collaborators [36] reported more than hundred natural products from actinomycetes, which exhibit anti-infective activities, and more than 70% of these metabolites were produced by *Streptomyces* strains. Moreover, approximately 40% of the described metabolites were synthesized by species inhabiting marine ecosystems. Antiviral properties were found for Antimycin A1a (Figure 1), a novel metabolite identified from the marine actinomycetes *Streptomyces kaviengensis*, isolated from the coast of New Ireland, Papua New Guinea. This compound (an antimycin A derivative) displays a significant activity against the Western Equine Encephalitis virus (WEEV), with IC_50_ value of less than 4 nM and selectivity index (SI), measured as the ratio of 50% cytotoxic (CC_50_) and inhibition (IC_50_) concentrations (SI = CC_50_/IC_50_), greater than 550 [17]. The encephalitic alphaviruses directly infect neurons resulting in central nervous system inflammation and neuronal destruction [40,41]. Similar to other antimycin A analogues, Antimycin A1a acts by inhibiting the cellular mitochondrial electron transport chain, and consequently suppressing *de novo* pyrimidine synthesis. Moreover, it is important to note that Antimycin A showed a broad spectrum of activity against a wide range of RNA viruses, including members of the *Togaviridae*, *Flaviviridae*, *Bunyaviridae*, *Picornaviridae*, and *Paramyxoviridae* families. These results indicated that marine actinomycetes are a promising source for antiviral drug discovery, and that the mitochondrial electron transport could be a possible target for the development of active antiviral compounds [17].

Butenolide analogs 1a, 1b, 2, 3, and 4, exhibiting anti-adenoviral property, were isolated from a marine *Streptomyces* strain (*Streptomyces* sp. AW28M48) collected from Vestfjorden, Norway [12]. Among these, the butenolide ketone 3 with a non-functionalized sidechain was the most promising anti-adenoviral agent with EC_50_ (half maximal effective concentration) value of 91 μM and no prominent cytotoxicity. The pre-incubation of the cells with the butenolide ketone 3 led to the complete blockage of viral replication, suggesting that this compound may act on a cellular target or process essential for viral replication. It was suggested that its prophylactic administration could prevent the viral infection, while, if administered after the infection, it could inhibit the spread of the infection to the cells not yet infected. However, the mechanism of action of the butenolide analog 3 is currently unknown, although it was demonstrated that the 2-furanone moiety in the structures of the isolated butenolides is important for the antiviral activity [12].

Antiviral activity was found in furan-2-yl acetate extracted from marine halophilic *Streptomyces* VITSDK1 spp., isolated from sediment samples collected at the Marakkanam coast (India). The extracted molecule exhibited activity against Fish Nodavirus (FNV), one of the most important viral pathogens of cultured marine fishes, responsible for huge economic losses. After exposure to furan-2-yl acetate (20 μg mL^−1^), the replication of Sahul Indian Grouper Eye (SIGE) cell lines infected by FNV was suppressed and the viral titer underwent a decline, from 4.3 to 2.45 log TCID_50_ mL^−1^. The results suggested a strong connection between the viral capsid protein inhibition and the decline in viral replication, although the mechanism of action has not yet been established [14].

In addition, we briefly report various bacterial extracts and mixture of compounds, such as exopolysaccharides (EPS), sulfoglycolipids, and lectins, which have been shown to possess antiviral activity. A marine EPS produced by *Pseudoalteromonas* sp. AM, isolated from a Red Sea sponge (Huraghada, Egypt), was characterized. It was reported to have antiviral activity against HSV type one (HSV-1), which led to the inhibition of 60.3% in the number of plaques after the treatment with 10% of the microbial EPS [15].

Tong and collaborators [42] showed that nine out of 38 microbial extracts obtained from marine microorganisms (including bacteria) isolated from Hawaiian waters had antiviral activities. Among the tested samples, three extracts, including the 482M(1) extract of bacterial origin, showed high inhibition against a broad-spectrum of viruses (Table 1), thus showing that they could be used as potential prophylactic agents, to prevent enveloped viruses infection, including HSV-1, Vesicular Stomatitis virus (VSV), and *Vaccinia virus*.

Bacteria isolated from marine sponges were tested to evaluate their potential antiviral activity against the Bovine Viral Diarrhea virus (BVDV), a surrogate model for antiviral assays for the development of agents against HCV [43,44], which causes chronic infections that can lead to liver cirrhosis and hepatocellular carcinoma in humans. Bacterial extracts obtained from the *Bacillus* sp. isolated from the sponge *Petromica citrina* gave the best results [45]. 

In addition, marine cyanobacteria represent a prolific source of natural products. The great biodiversity of cyanobacteria and of produced secondary metabolites assures that these microorganisms are able to produce a large array of bioactive molecules, ranging from sulfo-glycolipids and lectins [46,47,48], to alkaloids, lipopeptides, macrolides, sulphated polysaccharides, and other molecules [49]. Various compounds from cyanobacteria are on the market or in clinical trials (https://www.midwestern.edu/departments/marinepharmacology/clinical-pipeline.xml). 

The tropical filamentous cyanobacterium *Trichodesmium erythraeum* has been demonstrated able to produce a wide array of aplysiatoxins. Aplysiatoxin and related bioactive molecules, such as oscillatoxins and nhatrangins, are polyketide toxins isolated from various cyanobacteria, including *Lyngbya majuscula*, *Schizothrix calcicola*, and *Oscillatoria nigroviridis* [50,51,52]. Among them, aplysiatoxin-related compounds, Debromoaplysiatoxin and 3-methoxydebromoaplysiatoxin (Figure 1) displayed anti-Chikungunya virus (CHIKV) effects at concentrations that resulted in minimal cytotoxicity [13]. The antiviral mechanism of action is probably to target a step in the CHIKV replication cycle that occurs after viral entry. 

*Spirulina platensis* (now *Arthrospira platensis*), a cyanobacterium able to live in both freshwater and marine water, produces a wide range of bioactive compounds with antifungal, antiprotozoal, antiviral and antibacterial activity [53,54,55]. Silva et al. [16] demonstrated that ethyl acetate extract, rich in sulphated polysaccharide, of *Leptolyngbya* sp. is active against two Influenza viruses, A(H1N1)pdm09-WT and A(H3N2)-WT, by inhibiting the neuraminidase activity and replication. The latter effect is probably due to molecules capable of inhibiting different stages of the viral replicative cycle or thanks to the ability to activate host cell restriction factors.

### 2.2. Marine Fungi

Marine fungi are a rich source of novel bioactive molecules, probably produced as defense mechanisms. They produce a large number of marine natural products with promising biomedical applications [38], and it is thought that some of these compounds have the potential to proceed in clinical trials for future development of new drugs [56,57,58].

The antiviral potential of molecules isolated from marine-derived fungi, was highlighted after the isolation of Stachyflin from *Stachybotrys* sp. RF-7260, that showed antiviral activity against Influenza A (H1N1) virus [59]. Until 2006, a limited number of antiviral compounds was identified and reviewed [56]. On the contrary, after that, a large number of molecules with promising antiviral activities against several viruses, were isolated from marine fungi (Table 2). Most of these bioactive molecules were reviewed by Moghadamtousi and collaborators [60]; *Aspergillus* sp., *Penicillium* sp., *Cladosporium* sp., *Stachybotrys* sp., and *Neosartorya* sp resulted as the most important marine fungi exploited for their antiviral potential.

**Figure 2:**
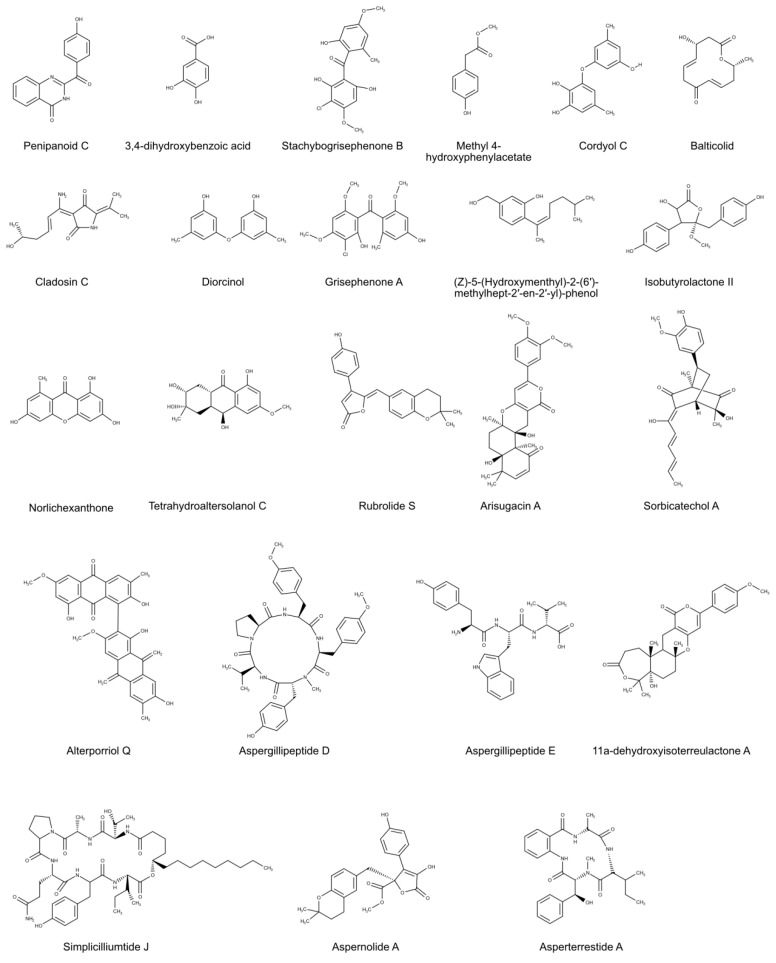
Structures of compounds isolated from marine fungi with antiviral activity.

Antiviral activity was found for three compounds, Stachybogrisephenone B, Grisephenone A, and 3,6,8-Trihydroxy-1-methylxanthone (Figure 2), isolated from the cultures of the sponge-derived fungus *Stachybotrys* sp. HH1 ZDDS1F1-2. These new sesquiterpenoid and xanthone derivatives showed inhibitory activities against in vitro replication of Enterovirus 71 (EV-71), that provokes acute neurological disease in children, with IC_50_ values of 30.1, 50.0 and 40.3 μM [18], suggesting that these compounds could be promising candidates for drug discovery for EV-71 and related viruses, such as Coxsackie virus (CVB3) [61].

Four novel compounds, including 11a-Dehydroxyisoterreulactone A, Arisugacin A, Isobutyrolactone II and Aspernolide A (Figure 2), were produced from a marine fungus, *Aspergillus terreus* SCSGAF0162, which was isolated from gorgonian corals *Echinogorgia aurantiaca* (South China Sea) [21]. These compounds showed antiviral activity against HSV-1, with a IC_50_ values of 33.38, 12.76, 62.08, and 68.16 μM, respectively.

Two marine-derived compounds with antiviral activity, Tetrahydroaltersolanol C and Alterporriol Q (Figure 2), were obtained from the marine-derived fungus *Alternaria* sp. ZJ-2008003, isolated from a *Sarcophyton* sp. soft coral (South China Sea). These compounds exhibited activity against the Porcine Reproductive and Respiratory Syndrome virus (PRRSV), that infects pigs and causes respiratory illness and a major problem in the reproduction of sows, with IC_50_ values of 65 and 39 μM, respectively [30].

A new compound, 2-(4-hydroxybenzoyl) quinazolin-4(3H)-one (Figure 2), and two known compounds, 2-(4-hydroxybenzyl) quinazolin-4(3H)-one and Methyl 4-hydroxyphenylacetate (Figure 2), showing antiviral activity, were isolated from the marine fungus *Penicillium oxalicum* 0312F1. The first compound exhibited moderate inhibitory activity, the other two compounds had potent inhibitory activity (with EC_50_ values 100.80 and 137.78 mg/mL, respectively) against Tobacco Mosaic virus (TMV), a virus that infects more than 400 assorted plant species, including cucumber, potato, tomato, and tobacco [32].

Potent inhibitory effect on the replication of TMV was also showed by two known compounds, AGI-B4 and 3,4-Dihydroxybenzoic acid (Figure 2), isolated from the culture of a marine-derived fungus *Neosartorya fischeri* 1008F1. Antiphytoviral test displayed effective activities, with IC_50_ 0.26 mmol L^−1^ and 0.63 mmol L^−1^, respectively. AGI-B4 also showed inhibition of the cell proliferation of human gastric cancer cell line SGC-7901 and hepatic cancer cells BEL-7404 [31].

A new 12-membered macrolide, Balticolid (Figure 2), was extracted from the fungal strain 222 belonging to the Ascomycota collected from the coast of the Greifswalder Bodden, Baltic Sea, Germany. At non-cytotoxic concentrations, Balticolid showed antiviral activity against HSV-1, with an IC_50_ value of 0.45 µM compared to 0.44 µM/aciclovir. Moreover, its structure was identified to be (3R,11R), (4E,8E)-3-hydroxy-11-methyloxacyclododeca -4, 8-diene -1, 7-dione [23].

Stachybotrins D is a new phenylspirodrimane with antiviral activities, produced from the marine fungus *Stachybotrys chartarum* MXH-X73, isolated from the sponge *Xestospongia testudinaris* collected from Xisha Island, China [62]. It acts by targeting reverse transcriptase, a fundamental enzyme in the Human Immunodeficiency virus (HIV) life cycle. This compound is a non-nucleoside reverse transcriptase inhibitor (NNRTI) of both wild-type HIV-1 (with EC_50_ value of 8.4 μM) and five NNRTI-resistant strains (with EC_50_ values ranging from 0.7- to 2.8-fold the value obtained against the wild-type virus) [19].

Rubrolide S (Figure 2) extracted from marine derived *Aspergillus terreus* OUCMDZ-1925, isolated from the viscera of the barracuda *Chelon haematocheilus* in the Yellow River estuary, exhibited activity against influenza A (H1N1) virus (IC_50_ value of 87.1 mM), comparable or superior to that of ribavirin (positive control), and weak cytotoxic effects on the K562 cell line [28]. 

Activities against Influenza virus A (H1N1) was also observed in two novel compounds isolated from the marine sediment-derived fungus *Penicillium chrysogenum* PJX-17, Sorbicatechols A (Figure 2) and B, with IC_50_ values of 85 and 113 μM, respectively [29].

Moreover, Cladosin C (Figure 2), isolated from the deep-sea derived fungus *Cladosporium sphaerospermum* 2005-01-E3 collected in the Pacific Ocean, showed mild activity against influenza A H1N1 virus, with an IC_50_ = 276 μM [26]. This activity is too weak to use cladosin C as drug, but it could be used as lead compound to develop new and more efficient drugs. Three known compounds extracted from the sponge-associated fungus *Aspergillus sydowii* ZSDS1-F6 (Xisha Islands of China), (*Z*)-5-(Hydroxymenthyl)-2-(60)-methylhept-20-en-20-yl)-phenol, Diorcinol, and Cordyol C (Figure 2), showed weak anti-H3N2 activity with IC_50_ values of 57.4, 66.5, and 78.5 mM, respectively [27].

Proteins and peptides from marine fungi have shown interesting antiviral activities, with minimal human toxicity and less side effects than synthetic drugs [58]. A new cyclic tetrapeptide, Asperterrestide A (Figure 2), isolated from the marine-derived fungus *Aspergillus terreus* SCSGAF0162, showed antiviral activity toward two Influenza A virus strains (H1N1 and H3N2) (with IC_50_ values of 15 and 8.1 μM, respectively), probably due to the presence of a 3-OH-N-CH3-Phe moiety which is rare in nature [25]. Moreover, Aspergillipeptides D–E (Figure 2), isolated from a marine gorgonian-derived fungus *Aspergillus* sp. SCSIO 41501, showed an evident antiviral effect versus HSV-1, with IC50 of 9.5 and 19.8 μM, respectively [22]. In addition, a new cyclic hexapeptide Simplicilliumtide J (Figure 2), together with known analogues Verlamelins A and B, were isolated from the deep-sea-derived fungal strain *Simplicillium obclavatum* EIODSF 020 and exhibited potent anti-HSV-1 activity (with IC_50_ values of 14.0, 16.7, and 15.6 μM, respectively), probably due to the presence of lactone linkage and a fatty acid chain moiety [24].

## 3. Marine Microalgae

Marine microalgae produce a huge number of metabolites with biological activity [63], including anticancer [64,65], anti-microbial [66], immunomodulatory [67], anti-diabetes [68], anti-tuberculosis [69], anti-epilepsy [70], anti-hypertensive, anti-atherosclerosis, anti-osteoporosis [71], and anti-inflammatory [20,72] activities. Even if microalgae are characterized by a huge biodiversity and amount of secondary metabolites, in the last 10 years, only a small number of studies reported antiviral activity of microalgal compounds (Table 3). 

**Figure 3:**
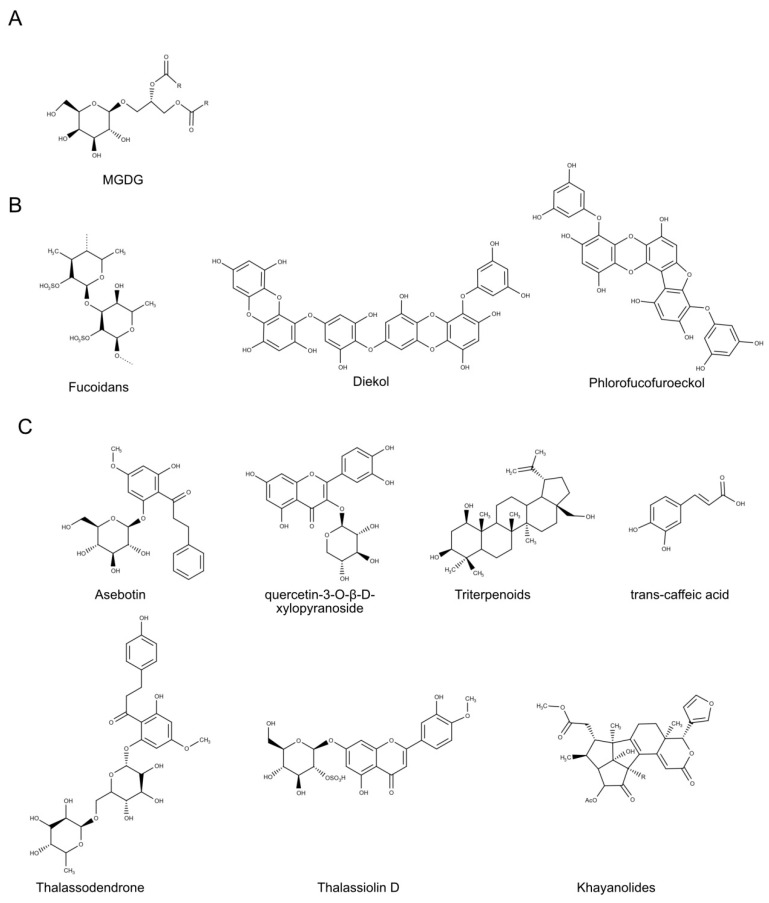
Compounds from microalgae (**A**), seaweeds (**B**), and seagrasses (**C**).

A monogalactosyl diacylglyceride (MGDG) (Figure 3A) isolated from the Trebouxiophyceae *Coccomyxa* sp KJ (IPOD FERM BP-22254) was found to be active against HSV. MGDG reduced viral activity in in vitro plaque assay of both HSV-1 (EC_50_ = 12–14 μg/mL) and Herpes Simplex virus 2 (HSV-2) (EC_50_ = 11 μg/mL) on African green monkey kidney cells (Vero cell line). Moreover, MGDG reduced the virus particle diameter of treated HSV-2 strain (untreated HSV-2 ranged from 272 to 308 nm, the virus particle diameter HSV-2 ranged from 66 to 118 nm) indicating changes in MGDG-treated virus particles both in the viral envelope and viral capsids. In in vivo experiment MGDG also reduced herpes symptom in treated mice [74].

EPS extracted from the Porphyridiophyceae *Porphyridium cruentum* have been found to reduce virus-induced cytopathogenicy of HSV, VSV and *Vaccinia virus* in in vitro assay on human erythroleukemia cell line (HEL), and the growth condition affected antiviral activity of extracted EPS [73]. A sulphated polysaccharide, derived from the Dinophyceae *Gyrodinium impudicum*, named p-KG03, was found to inhibit influenza A virus infection. In order to test antiviral activity of p-KG03, Madin-Darby Canine Kidney (MDCK) cells were infected with different strains of influenza A virus, H1N1 PR8, H1N1 Tw, and H3N2 and then treated in the presence of the sulphated polysaccharide. p-KG03 reduced plaque formation (EC_50_ were 0.48 µg/mL versus H1N1 PR8 strain; 0.19 µg/mL versus H1N1 Tw strain; 0.22 µg/mL versus H3N2). p-KG03 also reduced the viral nucleoprotein (NP) accumulation into the nucleus of MDCK cells [77]. Santoyo et al. [62] also found antiviral activity of polysaccharide extracts from the Chlorophyceae *Dunaliella salina*. Polysaccharides-rich extract of *Dunaniella salina* was tested to evaluate antiviral activity against HSV-1. Plaque formation assay using Vero cells showed antiviral activity of polysaccharides-rich extract with an EC_50_ of 85.34 µg/mL.

Marennine-like pigment from the Bacillariophycea *Haslea karadagensis* has been found to be active against HSV [75,76]. Marennine is the responsible of the greening effect on oyster [76] and two different forms of the marennine pigment have been found. Intracellular (IMn) and extracellular (EMn) marennine, the two forms of marennine, were different both in their molecular weights and in spectroscopic characteristics [75]. Marennine antiviral activity was tested by evaluating HSV-1 virus-induced cytopathogenicy [76] and viral titer [75] on Vero cells. Marennine displayed effective anti-herpetic activity, IMn and EMn forms reduced viral titer with similar EC_50_ values, 24 and 27 µg/mL, respectively [75]. On the contrary, EMn affected cytopathogenicy in a more efficient manner respect to IMn form (EC50 values were 23 µg/mL and 62 μg/mL, respectively) [76].

In addition, Silva et al. [16] tested crude organic extracts of the Dinophyceae *Symbiodinium* sp., the Raphidophyceae *Chattonella* sp. and Bacillariophyceae *Nanofrustulum shiloi* against Influenza A virus. The organic extracts were able to inhibit viral replication and infectivity of influenza A virus, both H1N1 and H3N1 strain.

## 4. Seaweeds

Seaweeds, generally classified into red algae (Rhodophyta), brown algae (Ochrophyta, Phaeophyceae) and green algae (Chlorophyta), are known for their potential activity against viral infections and, for this reason, applied in the formulation of medicated feeds for fish and invertebrates. Griffithsin, a protein isolated for the first time from an aqueous extract of the red alga *Griffithsia* sp. [78], is the only compound from macroalgae which reached clinical trials. Griffithsin is a lectin of 121 amino acids which has demonstrated in vitro and in vivo antiviral activity with minimum host toxicity against a variety of clinically relevant enveloped viruses (as reviewed by [79] and actually in clinical trials for HIV prevention (https://www.midwestern.edu/departments/marinepharmacology/clinical-pipeline.xml)). Most tests on macroalgae were aimed at defining their activity against HSV and HIV viruses [80], but more recently studies also concentrated on the activity against Influenza virus (Table 4) [81]. 

Studies on the brown alga *Eckolina cava* (Laminariaceae) report several biological properties, such as antioxidant [89], anticancer [90] and anti-inflammatory [91] properties. Ryu et al. [84] isolated the phlorotannin phlorofucofuroeckol (Figure 3B) from *E. cava*. Phlorofucofuroeckol has been found to inhibit neuraminidases activity of different Influenza A virus strains, H1N1, H3N2 and H9N2, with IC_50_ of 14.7, 20.7 and 22.7 μM, respectively. In addition, the phlorotannins diekol (Figure 3B) from *E. cava* showed inhibitory effects on the cell-free cleavage activity of SARS-CoV 3CL^pro^ (a chymotrypsin-like cysteine protease essential for severe acute respiratory syndrome coronavirus replication, SARS-CoV [92]), with IC_50_ of 2.7 μM [87]. 

Fish are subjected to several viral infections also in aquaculture practices, such as the viral Infectious Salmon Anemia (ISA) occasionally spreading over vast areas [93]. Vaccines or probiotics have been applied to attempt a reduction of economic damages. However, red seaweeds have been proposed and used as a feed ingredient for their antiviral activity. Results [85] on *Graciliaria chilensis* added to the diet of fish in concentrations as low as 10% demonstrated a clear antiviral activity salmon anemia virus. In addition, macroalgae may contain adjuvant compounds supporting the antiviral activity of other substances. For example, some algae are suitable as feed additives for anti-ISA virus, due to the presence of macro- and micronutrients such as silicon, taurine (44.9% higher content when compared to fish meal), eicosapentaenoic acid (EPA; in the case of lyophilized *Pyropia columbina*), and palmitic acid (in lyophilized *G. chilensis*). These nutrients, largely present in various macroalgae, play important roles in the immune system of vertebrates. Taurine, for example, is a strong antioxidant [94] and protects tissues against oxidative damage. Silicons triggers lymphocyte proliferation and modulate immune function through arginine [95]. The interaction between silicon and arginine affects immune functions, while a silicon deficiency weakens the proliferation of lymphocites. Polyunsaturated fatty acids, instead, are important components of cell membranes and dietary requirements can only be met with long-chain fatty acids docosahexaenoic acid (DHA) and EPA [96]. These fatty acids are precursors of eicosanoids, prostaglandins, and leukotrienes involved in the immune and inflammatory responses of fishes [97].

In addition White Spot Syndrome virus (WSSV), a pathogen causing a severe epidemic disease in shrimp [98], has emerged as problem in aquaculture. Declarador et al. [88] supplemented shrimp (*Penaeus monodon*) diet with the sulfated polysaccharide (SP) ulvan from *Ulva* sp and *Eteromorpha* sp. Ulvan had immunostimulatory activity against WSSV in juvenile *P. monodon*.

The SP ulvan, from the green seaweed *Ulva clathrata*, and its mixture with a fucoidan (SP from *Cladosiphon okamuranus*), were also found to have antiviral effects against the Newcastle Disease Virus (NDV) which causes morbidity in poultry [86]. Ulvan antiviral activity was tested using syncytia formation, exhibiting an IC_50_ of 0.1 μg/mL. It inhibited cell–cell fusion via a direct effect on the F0 protein but did not show any virucidal effect. Its combination with fucoidan had a reduced activity.

Brown algae, such as *Sargassum naozhouense,* have been used in Chinese medicine as antiviral drugs as well. S. *naozhouense* also contains various bioactive polysaccharides [83] against viruses, including HSV. *Sargassum* polysaccharides showed strong antiviral activity against HSV-1 strain F at ≥12.5 μg/mL (EC_50_ = 8.92 μg/mL). In order to compare antiviral potential of the polysaccharides, Peng et al. [83] used the antiviral drug Acyclovir (ACV) as a positive control and demonstrated that S. *naozhouense* extracts conferred more than 75% cellular protection at 20 μg/mL. Similarly, fractions extracted by various macroalgae collected along Brazilian coasts, containing glycolipids, exhibited potent antiviral activity against HSV-1-ACV susceptible (ACVs) and HSV-1-ACV resistant (ACVr) and presented low toxicity [80]. In particular, Phaeophyta (brown algae) produce several polysaccharides, as alginates, laminarans, and fucoidans (Figure 3B) [99,100]. Fucoidans, found in seaweeds [101,102,103,104,105,106,107], received a lot of attention due to their different antiviral activities [101,108]. Sulfated fucoidans from *Saccharina latissima* appeared to be responsible of the inhibitory effect on various viruses such as HSV-1 and CVB3 [109]. SPs from three seaweeds (*Grateloupia filicina*, *Ulva pertusa,* and *Sargassum qingdaoense*, i.e., Rhodophyta, Chlorophyta, and Ochrophyta, respectively) had immunomodulatory activity both in vitro and in vivo, on Kunming mice model, against Avian influenza virus (AIV). *G. filicina* SP exhibited the strongest anti-AIV activity [81]. Finally, polysaccharides from *Sargassum naozhouense* (mainly alginates and fucoidan) exhibited strong antiviral activity against HSV-1 in vitro with EC_50_ of 8.92 μg/mL [83].

## 5. Marine Plants

In this section we consider marine plants both seagrasses and mangrove. Seagrasses are angiosperms (flowering plants), evolved from terrestrial plants which have adapted to live in marine environments [110,111], and that live fully submersed in the sea [112]. Seagrasses can form extensive meadows distributed along temperate and tropical regions [113], influencing oxygen and carbon fluctuations in coastal areas [114], whose physiology and population structure have been shown to be influenced by biotic and abiotic stressors, including human effects and global changes [115,116,117]. Seagrasses, besides having an important ecological role [118], have also been used as traditional medicine [119]. Mangrove forests are composed by halophytic plants, and are mainly distributed in the tropical and subtropical regions [120,121]. The mangroves belonging to the genus *Sonneratia* (family Sonneratiaceae) have been used as traditional medicines for the treatment of several diseases [122].

### 5.1. Seagrasses

In the last decade, only a few numbers of compounds from seagrasses has been found to possess antiviral activity (Table 5). Two different studies find out antiviral properties of *Thalassodendtron ciliatum*. *T. ciliatum* is a common seagrass in the Red Sea, Tropical Indo-Pacific regions, Temperate Southern Ocean, the western part of Indian Ocean [123]. Thalassodendrone (6′-*O*-rhamnosyl-(1‴→6″)-glucopyranosyl asebogenin) (Figure 3C) has been reported to possess anti Influenza A virus activity. In order to evaluate the antiviral activity, MDCK cells were infected with Influenza A viruses and then treated in the presence of Thalassodendrone. Antiviral activity was reported as reduction of virus-induced cytopathogenicy. Thalassodendrone reduced cytopathogenicy with an IC_50_ of 1.96 μg/mL [124]. The phenolic compounds asebotin (2′,4,6′-trihydroxy-4′-methoxydihydrochalcone 2′-*O*-β-d-glucopyranoside), quercetin-3-*O*-β-d-xylopyranoside and *trans*-caffeic acid (Figure 3C) isolated from the same seagrass showed viral activity percentage reduction (96%, 70%, and 53% respectively) by plaque formation assay against HSV-1 at 2 mM [125].

Hawas et al. [126] isolated Thalassiolin D (diosmetin 7-*O*-β-glucoside-2″-sulphate) (Figure 3C), a flavone *O*-glucoside sulphate, from the seagrass *Thalassia hemprichii. Thalassia* sp. has already been reported to be source of various flavonoids that displayed wide range of biological properties, such as antibacterial [131] and anti-oxidative and skin-regenerating activities [132]. *T. hemprichii* is typically from tropical Indo-Pacific regions and Red Sea [123]. Thalassiolin D has been found to inhibit HCV protease, in in vitro assay, with an IC_50_ of 16 μM [126]. 

Antiviral activity of the polyphenol complex (PPC) from seagrasses of the Zosteraceae family was also studied [130]. Zosteracea species have been found to be widely distributed along different regions, such as temperate North Atlantic, temperate North Pacific, temperate Southern Ocean and Mediterranean [123]. Polyphenol complex from Zosteraceae mainly consists of rosmarinic acid, luteolin, and luteolin disulfate [133]. The polyphenol complex was tested against highly pathogenic strain of the Tick-borne encephalitis (TBE) virus of the Far-Eastern subtype Dalinegorsk (Dal), on porcine embryo kidney (SPEV) cells. Highest viral titer reduction was found in viral particles pretreatment in the presence of PPC (IC_50_ 80.8 μg/mL), when applied at the early stage of virus penetration PPC reduce virus titer with an IC_50_ > 100 μg/mL. Moreover, no significant reduction in virus titer was observed when SPEV cells were pretreated in the presence of PPC [130].

### 5.2. Mangroves

Several types of compounds have been found in mangroves (Sonneratiaceae) such as flavonoids, aromatic compounds, steroids, triterpenoids and alkaloids [134], and they have shown antioxidant [135] and cytotoxic activities [136]. The mangrove *Sonneratia hainanensis*, typical from Chinese coasts, has been found to possess several dimeric alkylresorcinols [127] (ARs, amphiphilic 1,3-dihydroxy-5-alkylbenzene homologues) that have shown to be a promising class of active secondary metabolites [137,138,139]. Two dimeric ARs, named integracins A and B, have been shown to have HIV-1 integrase inhibitory activities, with IC_50_ values of 3.2 and 6.1 μM, respectively [127]. Gong et al. [129] tested antiviral activity of triterpenoids isolated from *Sonneratia paracaseolaris,* a mangrove typically from China [140]. The isolated compound Paracaseolin A (1b,3b-dihydroxy botulin) was tested against influenza A H1N1 virus and the inhibition of viral activity was evaluated by cytopathic effect of the virus on MDCK cells. Paracaseolin A inhibited viral cytopathic activity with an IC_50_ value of 28.4 μg/mL. *Xylocarpus moluccensis* (Meliaceae) is a common mangrove from South Thailand [128], that produce several limonoids (modified tetranortriterpenoids) [141,142]. Khayanolides (Figure 3C), a class of limonoids, were isolated from the mangrove *X. moluccensis*. Three isolated khayanolides, named Thaixylomolins I, K, and M, exhibited anti-H1N1 activities, with IC_50_ values of 77.1, 113.5, and 121.5 μM, respectively [128]. Even if a huge number of secondary metabolites have been extracted from mangroves [119,122,125], only a small number of studies reported antiviral compounds (Table 5).

## 6. Marine Invertebrates

So far, lots of antiviral agents have been described from marine invertebrates [9,17,113,143,144,145,146], where the most promising organisms are represented by marine sponges [147]. Despite the numbers of antivirals found from marine invertebrates, only a few of them are on clinical trials or have been approved for drug marketing [148]. The antiviral compounds described in the last decade are listed in Table 6, Table 7, Table 8, Table 9, Table 10 and Table 11.

### 6.1. Sponges

**Figure 4:**
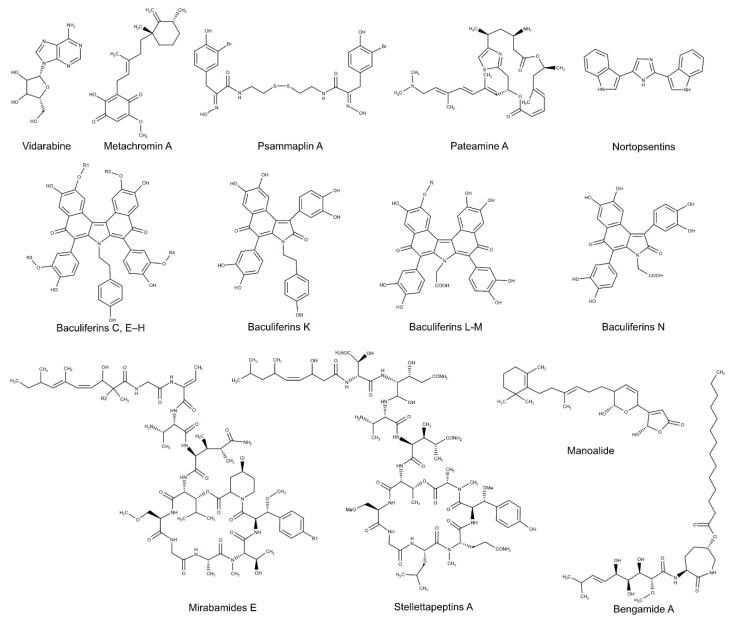
Compounds from sponge.

Since the first sponge-derived antiviral drug, Ara-A (Figure 4), was discovered as an anti-HSV-1, KOS strain agent [198,199], a great interest was addressed to marine sponges for exploring new pharmaceuticals [147,200]. Palem et al. [158] evaluated an anti HSV-1 activity in the sponge-derived alkaloid, Manzamine A, firstly isolated by Sakai et al. [201] as an antitumor compound. To test the antiviral capability, a recombinant virus, expressing the enhanced green fluorescent protein (EGFP), HSV-1 EGFP, was used to infect Seruminstitut Rabbit Cornea (SIRC) cells. The results showed a reduction of GFP expressing SIRC cells in those treated with manzamine A (1 μM) and, in addition, the viral release, quantified by a plaque assay, was reduced by 10^11^-fold. Moreover, the *n*-butanol fraction (BF), the halistanol-enriched fraction (TSH fraction) and the TSH isolated compounds halistanol sulfate and halistanol sulfate C, obtained from the crude extract of *Petromica citrina*, were tested against HSV-1 virus. The TSH fraction was the most active on HSV-1 replication (SI = 15.33) in comparison to halistanol sulfate (SI = 2.46) and halistanol sulfate C (SI = 1.95). Since a good synergism was detected between halistanol sulfate and halistanol sulfate C, the anti-HSV-1 efficacy of TSH fraction probably could depend on the cooperation of the two halistanol sulfates [159]. 

Fan et al. [154] demonstrated a strong activity of several pyrrole alkaloids from the Chinese marine sponge *Iotrochota baculifera* against another group of viruses, the HIV type 1 (HIV1). Thanks to in vitro tests on the HIV-1 susceptible MT4 and the single life cycle MAGI cells (HeLa-CD4-LTR-b-gal cell line), the mechanism of these compounds, Baculiferins C, E–H and K–N, (Figure 4) was described as a strong interaction with three main targets: (i) HIV-1 trans-membrane protein (gp41), (ii) HIV-1 viral infectivity factor (Vif) and (iii) human innate intracellular anti-viral factor (APOBEC3G). SPs, isolated from three sponges, *Erylus discophorus*, *Cliona celata* and *Stelletta* sp., were also tested for anti-HIV-1 activity. In addition, four depsipeptides (Mirabamides E–H, Figure 4) isolated from species belonging to *Stelletta* genus, showed promising results. Increasing concentrations of Mirabamides E-H displayed a strong inhibition of viral replication in genital epithelial cell model (TZM-bl target cells, constitutively expressing CD4 and CCR5 HIV-1 receptors), with IC_50_ values of 40 nM for Mirabamide H, 65 nM for Mirabamides F–G) and 120 nM for Mirabamide E [156]. Additional depsipeptides, Stellettapeptins A-B (Figure 4), isolated from the same genera were also analyzed. Bioactivity testing on human T-cell line (CEM-SS) infected with HIV-1_RF_ virus was performed and a significant reduction of the HIV-1 cytopathic effect (EC_50_ = 23 and 27 nM) was observed in samples treated with the sponge-derived depsipeptides [157]. Furthermore, Yu et al. [153] found an anti-HIV-1 activity in several aaptamine alkaloids, isolated from *Aaptos aaptos* species. Among eight compounds tested, two of them revealed a good inhibition of HIV-1 (77.3–88%) at 10 μM. Recently, Bengamide A (Figure 4), firstly isolated by Quinoa et al. [202] from *Jaspis* cf. *coriacea*, has been shown to be potent anti-HIV-1 agent. In particular, treatments of CD4+ T-lymphocyte cell line encoding for a GFP reporter and infected with HIV-1_NL4.3_, induced a good inhibition of viral replication, with EC_50_ values of 0.015 μM [155]. Moreover, HIV-1 LTR NF-κB response elements were demonstrated to be necessary for Bengamide A activity, since treated Jurkat T cells coding for an LTR-driven luciferase construct and mutated NF-κB elements (pLTRmNF-κB-RL) were less responsive in terms of luciferase activity (EC_50_ > 3 μM) [155].

Several sponge-derived compounds were efficient antiviral agents against human hepatitis such as, HCV, Hepatitis B virus (HBV) and Hepatitis A virus (HAV). A sesterterpenoid antibiotic isolated from the sponge *Luffariella variabilis* [203], Manoalide (Figure 4), was investigated for HCV activity [151]. This compound acted as a potent inhibitor of the NS3 RNA helicase and NTPase activity (IC_50_ of 15 and 70 μM, respectively,) essential for the replication of viral genomic RNA. The results suggested that manoalide was able to bind a conserved helicase motif of the NS3 viral protein, interfering with its ATPase function [151]. The same authors found an additional HCV-NS3 antagonist, the sponge-derived Psammaplin A (Figure 4) [152,204]. In particular, they demonstrated that, this brominated tyrosine derivative, blocks the NS3 RNA helicase (IC_50_ = 17 μM) and ATPase (IC_50_ = 32 μM) activities but, although the viral replication was inhibited in HCV replicon cells, a low SI index was calculated. For this reason, a possible chemical modification was suggested in order to improve the anti-HCV efficacy [152]. An anti-HCV activity was also evaluated in the extracts and fractions of two marine sponges, *Homaxinella tanitai* and *Microxina subtilis*. 

A group of diverse samples collected from the coral reefs of Indonesia were screened against the HBV core promoter activity, which is fundamental for viral replication [150]. More specifically, two polybrominated diphenyl ethers (PBDEs), isolated from *Dysidea* sp., were found potent inhibitors when incubated with pGL4.18 CURS_BC_AeUS transfected Huh7 cells encoding for the HBV promoter regions. In addition, the anti-HBV activity was also confirmed by Real Time-qPCR approach and MTS assay [150]. To extend their knowledge on sponge-derived anti-HBV agents, Yamashita et al. [149] further investigated several compounds for identifying those able to inhibit the HBV core promoter. Among fifteen terpenes tested, metachromin A (Figure 4), a merosesquiterpene purified from *Dactylospongia metachromia*, was considered a good antiviral agent by inhibiting the core promoter and viral replication with EC_50_ value of 0.8 μM (SI = 19.6). Studying the anti-HBV activity of metachromin A derivatives and analogues, the hydroquinone group and the double bonds of C-5 and C-9 were found essential for HBV core promoter blocking [149]

González-Almela et al. [160] focused on a diverse family of viruses, the Sindbis virus (SINV), transmitted by mosquitoes causing the sindbis fever in humans. Pateamine A (PatA) (Figure 4), a natural compound synthetized by the sponge *Mycale* sp., was a potent suppressor of SINV subgenomic mRNA (sgmRNA) translation by targeting eIF4A complex, composed of the cap-binding factor eIF4E, the helicase and ATPase enzyme eIF4A and the scaffolding protein IF4G. Particularly, Baby Hamster Kidney (BHK) fibroblast cells were infected with SINV virus and treated with PatA (100 nM). Immunoblotting using specific monoclonal antibodies revealed that PatA inhibited the synthesis of early nonstructural proteins (nsP1 and nsP2), leading to the block of viral RNA replication and transcription. However, SINV virus was sensitive to PatA at early stages of viral infection, while a significant decrease of PatA efficacy was observed when treatment was applied during late viral processes [160]. In a recent study, a group of alkaloids, called nortopsentins (Figure 4), isolated from *Spongosorites ruetzleri*, displayed in vitro and in vivo antiviral activity against TMV and some of them were more active than the plant virucide ribavirin at 500 μg/mL. Since chemical modifications were able to compromise or revert the antiviral activity, these sponge-derived alkaloids were demonstrated to be very sensitive to substituents [161].

In addition, several extracts from sponges have been found to have antiviral activity. Methanol extracts and *n*-butanol fractions of nine sponges were analyzed for anti-HSV-1 activity and the most promising samples were those obtained from the *Haliclona* (*Halichoclona*) sp. and *P. citrina* species [205]. The organic extracts of *Aka cachacrouense*, *Niphates erecta,* and *Dragmacidon reticulatum*, were reported to possess moderate activity against HSV1 [206]. The ethyl acetate extracts of *H. tanitai* and *M. subtilis* showed antiviral activities against HCV [207], while, the crude extracts of *Callyspongia crassa* and *Callyspongia siphonella* [208], and of *Grayella cyathophora* revealed anti-HAV activity [209].

### 6.2. Mollusks

Several antiviral compounds, especially hemocyanins [210], were described from mollusks and many of them have diverse mechanisms of action against human pathogens [211,212]. The antimicrobial peptide, Myticin class C (Myt C), mostly found in the hemocytes of the mussel *Mytilus galloprovincialis*, was tested for its activity against the Viral Hemorrhagic Septicemia virus (VHSV) and Infectious pancreatic necrosis virus (IPNV) [168]. CHSE-214 (Chinook salmon embryo) cells were transfected with a plasmid encoding for MytC-eGFP sequences and then infected with VHSV virus. The viral replication was evaluated by Real Time-qPCR using specific primer for the N protein of VHSV and for the segment A of the IPNV genome. The results showed that a significant inhibition of VHSV replication (about 75–85%) was induced by Myt C, while no significant effects were detected for IPNV virus [168]. The hemolymph of *M. galloprovincialis* and Myt C peptide also inhibited the replication of the ostreid herpesvirus 1 (OsHV-1) in the hemocytes of oysters [167]. In addition, when Myt C was modified or nanoencapsulated, a potent HSV-1/HSV-2 was found. In fact, significant SI values (>8.21 for HSV-1 and >10.5 for HSV-2) were measured in treatments with the modified Myt-Tat, which was supplied with 13 additional C-terminal amino acid residues corresponding to the HIV-1 cell-penetrating peptide (CPP). Significative SI values (>7.69 and >8.32 for HSV-1 and HSV-2, respectively) obtained with encapsulated Myt C into commercially nanovesicles confirmed that the antiviral activity depended on the efficient penetration inside the viral cells [167].

Dang et al. [213] used the hemolymph and peptide fractions, from the abalone, *Haliotis laevigata*, for an antiviral screening against HSV-1. Vero cells in vitro tests revealed that the abalone hemolymph significantly reduced the viral plaque number and size [213]. The hemolymph serum and three hemocyanin fractions (R1, R2, and R3) of the other abalone species, *Haliotis rubra*, were tested against HSV-1 infection. The antiviral efficacy of the three fractions was higher than the total serum with SI values of 9.9 (R1), 12 (R2), 9.7 (R3), and 2.6 (serum). Moreover, hemocyanin directly binds the viral surface through the glycoproteins gD, gB, and gC, inhibiting its entry in the host cell [164]. Since Zanjani et al. [214] found that a synthetic formulation of hemocyanin with the disaccharide trehalose was stable and with a long shelf life, being this abalone-derived compound a good candidate for pharmacological applications. The anti-HSV-1 capability of the *H. rubra* was influenced by water temperature, since the highest activity was found in February (26.5 °C; plaque reduction = 63.76%), while the lowest in September (12.5 °C; plaque reduction = 46.04%). These data were confirmed by in vitro experiments, which demonstrated a greater activity at 24 °C (plaque reduction = 72.5%) than 18 °C (plaque reduction = 40%) after seven days of incubation [215]. A comparative study on the abalones *H. laevigata*, *H. rubra* and their hybrid was also performed. Plaque reduction assays in HSV-1 infected Vero cells treated with the hemolymph showed no significant differences between *H. laevigata*, *H. rubra* and the hybrid. Interestingly, a higher anti-HSV-1 activity was observed in the hemolymph from wild individuals than farmed ones [166].

A different work published by Green et al. [165], described the anti-HSV-1 activity of protein fractions obtained from the hemolymph of the Pacific oyster *Crassotea gigas*. The most active fraction, evaluated by Vero cells plaque assay, was then analyzed through LC/MS-MS approaches, which showed the presence of the typical glycoproteins called cavortins. The Hemocyanin RvH extracted from another mollusc, the marine snail *Rapana venosa*, was investigated for its activity against Epstein-Barr virus (EBV) on the lymphoblastoid B-cells Raji line. PCR approaches showed that RvH reduced the number of genomic equivalents of EBV DNA with 50% inhibitory dose (ID_50_) of 1 μg/mL [162]. Two structural subunits (RvH1 and RvH2) of *R. venosa* hemocyanin, including the functional units RvH1-a and RvH2-e, were also active against EBV virus, reducing the viral replication (at 1, 10, and 100 μg/mL) in lymphoblastoid cells of B-phenotype (Raji, B95-8 and Namalwa) [163].

### 6.3. Cnidarians

**Figure 5:**
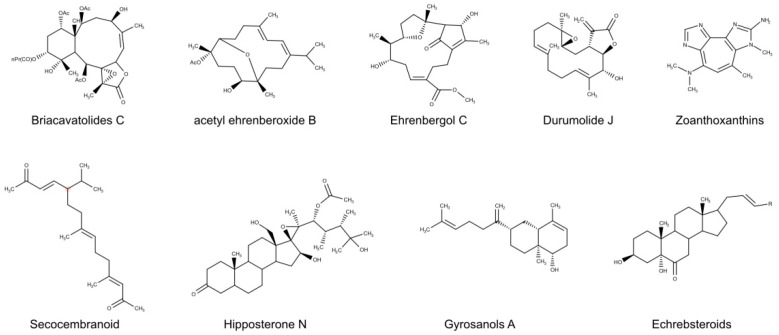
Compounds from cnidarians.

Among Antozoa, soft corals, especially those belonging to the Alcyoniidae family, are recognized as a rich source of a large variety of bioactive molecules, ranging from sesquiterpenes to diterpenes, polyhydroxylated steroids, and polyamine metabolites [38,216], with cytotoxic, anti-inflammatory and antimicrobial activities [216]. Studies carried out on *Sinularia gyrosa* led to the discovery of interesting antiviral compounds, such as an unusual norcembrane-type diterpenoid and three new gyrosanols [216]. Two of these gyrosanols (Gyrosanols A, Figure 5, and B), structurally related to compounds identified in other Antozoans [217,218,219,220] showed antiviral activity against Human cytomegalovirus (HCMV) with an IC_50_ value of 6,6 μM [174]. In addition, a Durumolide J-like (Figure 5) compound identified in *Lobophytum durum* and a Secocembranoid (Figure 5) isolated from *Lobophytum crassum* exhibited significant antiviral activity against HCMV with IC_50_ values of 14.3 µM [172] and 12.7 µM [176], respectively. *Lobophyton* is not the only soft coral able to produce anti-HCMV compounds. In fact, the acetone extract of *Sarcophyton ehrenbergi*, sampled along Taiwan shores, was found rich in antiviral diterpenoids and two of them, called Ehrenbergol C and Ehrenberoxide B (Figure 5), demonstrated an antiviral activity toward HCMV with IC_50_ values of 52.8 and 21.9 µM, respectively [173]. An activity against HCMV was also observed from the polyoxygenated steroid Hipposterone N (Figure 5), isolated from the wide distributed gorgonian *Isis hippuris* (EC_50_ values of 6.0 μg/mL) [175] and in Briacavatolides C (Figure 5) and F, two briarane-type diterpenoids isolated from acetone extract of *Briareum excavatum*, which showed IC_50_ of 18 µM (Briacavatolides C) and a 50% effective dose (ED_50_) of 22 µM (Briacavatolides F) [170,171].

*Sinularia* and *Sarcophyton* species produced an interesting bioactive compound with antiviral properties against various influenza strains. A polyhydroxylated sterol together with three new ceramide derivatives were isolated from *Sinularia candidula*, a soft coral living in the Egyptian Red Sea. These compounds exhibited selective antiviral activity against the orthomyxovirus of the avian influenza H5N1, revealed by plaque reduction assay in MDCK cells [178]. Activity against orthomyxovirus (H1N1) were displayed from two polyhydroxylated steroids produced by *Sarcophyton* sp. collected in the South China Sea, with IC_50_ values of 19.6, 38.6, and 73.06 µM, in comparison with the positive control ribavirin (IC_50_ = 102.21 µM) [179].

*Eunicea* and *Pseudopterogorgia* gorgonian species are rich sources of bioactive compounds, such as sesquiterpenes, cembranoid, and fuscoside diterpenes, which showed an antiviral activity through an unrecognized mechanism of action in addition to the well-known anti-inflammatory and anti-bacterial potential. Investigations on *Echinogorgia rebekka* led to the identification of three echrebsteroids (Figure 5), showing moderate (Echrebsteroid A, IC_50_ = 0.78 µM) and strong antiviral activity (Echrebsteroids B and C, IC_50_ = 0.19 µM) against the Respiratory syncytial virus (RSV) in human laryngeal carcinoma (Hep-2) cells [180]. From a strictly related species, *Echinogorgia pseudossapo*, anti-HSV-1 compounds were also isolated and characterized. In particular, the compound pseudozoanthoxanthins III and another zoanthoxanthin alkaloid (Figure 5) displayed interesting activities against HSV-1 [177]. Moreover, anti-HSV-1 activity of the extracts from three additional gorgonian species, *Eunicea succinea*, *Eunicea fusca,* and *Pseudopterogorgia elisabethae* was evaluated. In particular, these diterpens-rich octocorals showed antiviral activity with IC_50_ values ranging from 50 to 62.5 µg/mL [206]. 

In addition, organic extracts of *Lobophyton microlobulatum* and *Sarcophyton auritum* showed anti-CHIKV activity [221], as well as *Sinularia kavarattiensis*, whose activity was probably due to a synergic effect of various norcembranoids and sesquiterpenoids (isolated in the biological active enriched chloroform extract) with various levels of inhibition on the CHIKV virus replicon in BHK21 cell line [169]. Finally, *Cassiopia andromeda* lipophilic fraction showed a potent inhibitory activity against HIV-1 protease [222]. In the same investigation, a moderate inhibition of HIV-1 protease was shown by the lipophilic fractions of the soft corals *Sinularia heterospiculata, Lithophyllum arboreum,* and *Sinularia maxima*. 

### 6.4. Crustaceans

Among crustaceans, several studies focused on potential antiviral agents against the White spot syndrome virus (WSSV), which is a viral pathogen causing a severe epidemic disease in farmed animals [98]. Several molecules, especially antimicrobial peptides (AMPs), involved in crustacean immune response, were considered as anti-WSSV agents. The hemocyte proteins (Sp-ALFs) from the mud crab, *Scylla paramamosain*, were found potent anti-WSSV compounds when tested in hematopoietic tissue (Hpt) cell cultures from the freshwater crayfish, *Cherax quandricarinatus*. In fact, Real Time-qPCR analyses revealed that the mRNA levels of an early gene involved in WSSV replication (IE1), significantly decreased when Hpt cells were infected with WSSV viruses pre-treated with Sp-ALFs proteins [184]. A similar work investigated the anti-WSSV capability of Scygonadin, an AMPs from the crub *S. paramamosain*. A recombinant peptide was firstly expressed in the yeast *Pichia pastoris* and then tested on Hpt cells with scygonadin at 25 µM or 50 µM. After 3 h of incubation with scygonadin-WSSV mixtures, a dose-dependent down-regulation of IE1 transcripts was detected [186]. Du et al. [185] described a novel Peroxinectin (PX) analog (Sp-PX) from the same crub species *S. paramamosain*, which showed antiviral properties. Gills injections of plasmids encoding for a WSSV sequence induced a strong up-regulation (about 10-fold increase) in the hemocytes (12 h post-injection, hpi), revealed by Real Time-qPCR analyses using specific primers for Sp-PX gene. Interestingly, Sp-PX was found down-regulated at 96 hpi, stimulating the hypothesis that Sp-PX played a crucial role in the crub immune response during the early stage of WSSV infection. Recently, the same authors further explored the role of a family of crustacean AMPs, called crustins. A recombinant crustin, Sp-Crus6, was pre-incubated with WSSV viruses and injected in *S. paramamosain* crub gills. As a result, a decrease of virion load was observed at 48, 72, and 96 hpi, detected by Real Time-qPCR amplification of WSSV fragment [182]. 

Another group of compounds, β-thymosin-repeat proteins (mjthm4, mjthm3, and mjthm2), firstly described in the freshwater crayfish *Procambarus clarkii* as antiviral agents released in response to WSSV infections [223], were also found from the shrimp *Marsupenaeus japonicus*. Real Time-qPCR revealed a significative up-regulation of β-thymosin transcripts in the hepatopancreas of shrimps injected with WSSV viruses, reaching the highest values at 6 hpi (3.28-, 3.54-, 3.71-fold) [188]. The single whey acidic protein (WAP) domain (SWD)-containing proteins, a family of AMPs, were studied from *Litopenaeus vannamei*, for its possible role in the antiviral immunity. In particular, RNA interference (iRNA) of a novel SWD (LvSWD3) protein accelerated the death of WSSV infected shrimps and significantly increased the viral load at 48 and 72 hpi in the shrimp muscle. To confirm the antiviral activity, a recombinant LvSWD3 was expressed in *Escherichia coli* hosts and then used to treat shrimps. As hypothesized by iRNA approach, the recombinant protein displayed antiviral capabilities, since a viral load reduction in the muscle of infected shrimps was observed [187]. A shrimp hemocyanin derived peptide, LvHcL48, was also evaluated in vitro and in vivo for its antiviral activity in *L. vannamei*. Treatments of hemocyte cultures with LvHcL48 peptide-WSSV mixtures significantly attenuated the transcription of two WSSV genes (*wsv069* and *wsv421*). These results were confirmed by in vivo experiments, demonstrating that WSSV pre-treated viruses with LvHcL48 peptides decreased the *wsv069/wsv421* mRNA levels at 6, 12, and 24 hpi compared to the control group (PBS + WSSV). In addition, Far-Western blotting assay on WSSV lysates demonstrated that LvHcL48 was able to interact to the viral envelope protein VP28 [183]. 

Antiviral activity of the crustacean-related compound, chitosan, on human Norovirus and enteric virus surrogates, plus Feline Calicivirus (FCV-F9), Murine Norovirus (MNV-1) and two bacteriophages (MS2 and phiX174) was also evaluated. Chitosan is a marine polysaccharide of crustacean’s exoskeleton with a potent antimicrobial agent against both Gram-negative and Gram-positive bacteria [224]. Plaque reduction assays, performed in host cells infected with virus/chitosan mixtures, revealed that chitosan was mostly active on MS2/phi X174 phages and FCV-F9, and, in some cases, the molecular weight (MW) of chitosan and medium pH strongly influenced the antiviral capability, suggesting that further studies were needed before proposing this polysaccharide for pharmacological applications [181].

### 6.5. Echinoderms

Marine organisms belonging to *Echinodermata* are also rich source of bioactive compounds although a low chemical diversity has been recorded compared to other phyla [38]. Echinoderm-derived natural products were mostly sulfated compounds that can be classified into two mayor groups, aromatics and saponins. In the last decade, saponins isolated from sea cucumbers are receiving a greater attention due to their interesting biological features [225,226]. Conversely, aromatic sulfated compounds were mostly reported in crinoids and ophiuroids as pigments deriving from anthraquinones or naphthoquinones [227].

The phospholipase A2 (AP-PLA2) from the sea star *Acanthaster planci* was evaluated for its anti-HIV-1 activity. In particular, treatments with AP-PLA2 significantly reduced the number of infected phytohemagglutinin-stimulated peripheral blood mononuclear cells (PBMC). Moreover, Real Time-qPCR and gel electrophoresis revealed that the expression of the HIV group-specific antigen (Gag) strongly decreased in treated PBMC cells. The decline of infection rates was also confirmed by immunofluorescence approaches [190]. 

**Figure 6:**
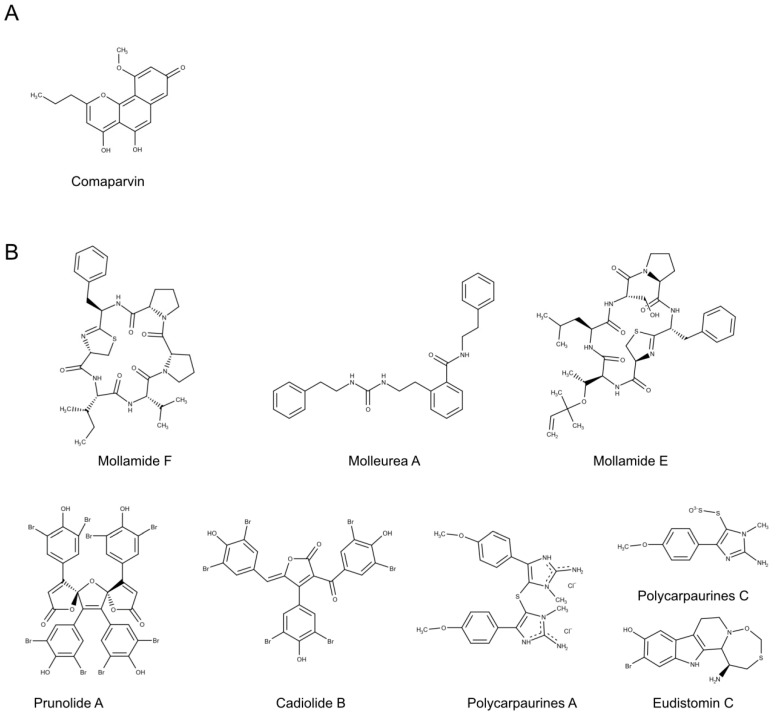
Compounds from echinoderms (**A**) and tunicates (**B**).

Seven known naphthopyrones and a novel pyrano[2,3-f]chromene were recently isolated from the water/ethanol extract of the Australian crinoid *Capillaster multiradiatus* and tested for HIV-1 potential. Among all compounds, comaparvin (Figure 6A) displayed the highest activity in HIV-1_NL4.3_ infected CEM-GXR cell lines, evaluated by GFP fluorescence after 72 h of treatment. Comaparvin showed EC_50_ values of 7.5 μM, while the other compounds reached EC_50_ values ranging from 14.5 to 25.5 μM [191].

A family of sulfated sterols isolated from cold water echinoderms together with their synthetic derivatives and analogues [228,229,230] were tested on HSV-1, HSV-2 and Pseudorabies virus (PrV) strains. Among the twelve sterols investigated, disodium 2b,3a-dihydroxy-6E-hydroximine-5a-cholestane-2,3-disulfate was the most effective, with a broad spectrum of action since viral plaques were significantly reduced in Vero cells infected with all viral strains (EC_50_ values of 16.5, 17.9, 17.2 μg/mL). Unfortunately, two hemorrhagic-fever-causing viruses, Junín virus (JUNV) and Dengue virus (DENV), for which no therapies are still available, the active sulfated steroid did not show any reduction, with higher EC_50_ values (>25 μg/mL) [193].

The acidic mucopolysaccharide (SJAMP) from another sea cucumber species, *Stichopus japonicus*, was tested in vivo for anti-HBV activity. HBV-DNA serum levels induced a dose-dependent reduction detected by Real Time-qPCR, when mice were injected with increasing concentrations of SJAMP. The histologic sections of liver tissues also showed a conspicuous number of inflammatory cells in the interstitium and a visible increase of neutrophilic leukocytes and vacuoles [189].

Moreover, extracts from echinoderms were found to possess antiviral activity. The anti-HIV-1 activity was observed in the methanol and diethyl ether extracts from the sea cucumber, Holothuria leucospilota [231]. The water extract of the sea cucumber Holothuria sp. was found active against HSV-1 virus [232]. Finally, anti-HSV-1 activity was also observed in hydrolysates and solvent extractions from the pharyngeal bulb and internal organs of Cucumaria frondosa [192].

### 6.6. Tunicates

The first ascidian metabolite, geranyl hydroquinone, isolated from *Aplidium* sp., displayed chemo-protective activity in leukemia, rous sarcoma and breast cancer treatments [233,234]. Since then, numerous new bioactive compounds, mostly anticancer agents, have been discovered from tunicates [235,236,237,238]. For instance, tunicate-derived compounds include trabectedin and its analogue lurbinectedin were clinically approved for antitumor therapies [239]. Regarding tunicates-derived antiviral products, few recent works were reported in literature. Two new cyclopeptides, mollamides E and F (Figure 6B), and a new tris-phenethyl urea, molleurea A (Figure 6B), from the methanol extract of *Didemnum molle*, were tested for HIV-1 activity. Mollamide F and molleurea A reduced viral replication in HIV-1 infected cells with IC_50_ values of 78 and 60 μM, respectively, whereas mollamide E was only active on HIV-1 integrase (IC_50_ = 39 μM) [195]. The crude extract and a furanone metabolite, rubrolide R, isolated from the ascidian *Synoicum*, together with the known compounds rubrolide A, cadiolide B and prunolide A (Figure 6B), were tested against the Japanese encephalitis virus (JEV). Among these compounds, prunolide A and cadiolide B, previously isolated from *Synoicum prunum* [240] and *Botryllus* sp. [241], showed antiviral properties in Vero cells against the JEV at 1.7 and 1.4 μM respectively [194]. A diverse group of alkaloids, polycarpaurines A and C (Figure 6B), isolated from *Polycarpa aurata* were evaluated against TMV virus and compared to the commercial virucide ribavirin. In vitro and in vivo experiments of polycarpines and synthetic derivatives displayed controversial outcomes, since the presence of sulfur groups increased the antiviral activity in cells, while the highest in vivo anti-TMV efficacies were recorded when tested compounds lacking in S atoms [197]. The mechanism of action of another antiviral agent, eudistomin C (EudiC) (Figure 6B), extracted from the New Zealand ascidian *Ritterella sigillinoides* [242] was described by Ota et al. [196]. The activity against HSV-1 and Polio vaccine Type I viruses was correlated to the block of protein translation by interacting to the uS11-containing small ribosomal subunit in yeast [196].

### 6.7. Other Invertebrates

Additional antiviral activities were also found from the peanut worm *Sipunculus nudus*. This marine organism, belonging to *Sipuncula* phylum, is considered by traditional Chinese medicine a rich source of beneficial compounds used to treat a variety of diseases and for anti-aging purposes. Su et al. [243] tested the water-soluble polysaccharides (SNP) extracted from *S. nudus* for antiviral activity against HBV virus in human hepatoblastoma cell lines (Hep-G2/2.15), having a stable HBV expression. Treatments with increasing concentrations of SNP compounds (1, 0.5, 0.25, and 0.13 mg/mL) significantly dropped down the expression of HBV-DNA and Hepatitis B surface antigen (HBsAg) after 48 h of incubation. In addition to antiviral evidences, the relative transcripts of pro-apoptotic genes increased the expression of pro-apoptotic proteins TNF-α, caspase-3, and Bax in dose- and time-dependent manner, confirming that these marine-derived compounds were also able to induce apoptotic events in Hep-G2/2.15 cells.

## 7. Marine Vertebrates

In the last 10 years, few studies reported antiviral activities from marine vertebrate, and all of them mainly focused on peptides from fishes. The mucosal tissue of the fish is where the first encounter between virus and host begins and several mechanisms act against pathogens in the mucus layer such as mucus shedding and reproduction, mucosal antibodies, and antiviral peptides [244,245,246]. Antifreeze peptides (AFPs) from marine polar fishes have high similarity to the AMPs, both in structural and physical-chemical properties [247]. A modified antifreeze peptides (AFPs) from marine polar fish *Pleuronectes americanus*, named *Pa*-MAP (multiple active peptides), has been shown to possess promising biological activities. In order to test antiviral properties of *Pa*-MAP, Vero cells were infected with HSV-1 or HSV-2, and treated with the linear peptide. *Pa*-MAP reduced viral titer in the media of treated cells and reduced virus titer of both HSV-1 and HSV-2 after infection of Vero cells. *Pa*-MAP induced 82% of HSV-1 titer reduction at a concentration of 45 μM and 90% of HSV-2 at 23 μM [248]. Further studies reported that *Pa*-MAP was able to damage viral particles thus affecting the viral adsorption [249].

PLA2 from the venom of red lionfish (*Pterois volitans*, *PV*) has been found to possess anti-HIV activity. *P. volitans* is a nocturanal predator species that have native habitats in the Indo-Pacific oceans [250]. *PV*-PLA2 was tested on simian retrovirus-2-infected human cell line A549 cells (SRV2-A549). Analysis of antiretroviral activity with were performed by Real Time-qPCR and cycle threshold (Ct) value was used to evaluate the number of viruses in the sample. *PV*-PLA2 has been shown to affect antiretroviral activity of SRV2, suggesting that *PV*-PLA2 can be a good anti-HIV drug candidate [251]. PLA2 from other source has been also found to have antiretroviral activity against HIV [190,252,253].

## 8. Conclusions

**Figure 7:**
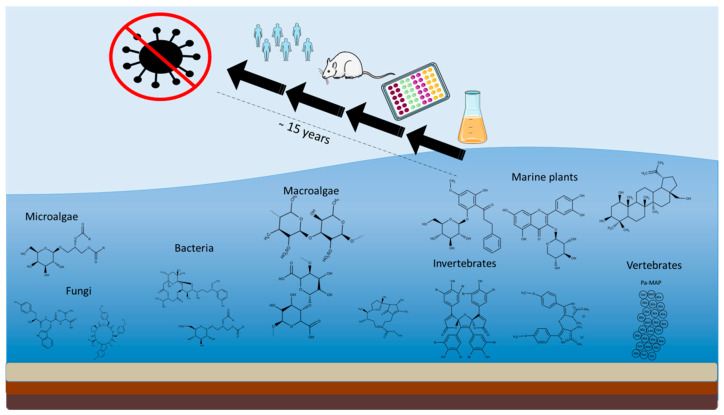
This figure shows time estimates for research and development of new Food and Drug Administration approved antiviral drugs and some examples of antiviral drugs from marine organisms.

Marine organisms are well-known to produce compounds with potential pharmaceutical applications [2]. This review show that marine organisms can produce a plethora of compounds with antiviral activities (i.e., against HIV, HSV, HHV, Influenza A virus, *Vaccinia virus*, SRV-2, HAV, HBV, HCV; EBV, Enterovirus, HCMV, JEV, TMV, PrV, WSSV, MS2, CHIKV, OsHV, SINV, TBE, PRRSV, EV-71, FIPV, MHV, BVDV, KHV, WEEV,). Different classes of compounds have been found, such as carbohydrates, exopolysaccharides, lipids, peptides, alkaloids, polyphenols, steroids, polyketides, terpenoids and zoanthoxanthins. However, until now only one marine derived compound with antiviral activity reached the market, Ara-A (against Herpes Simplex Virus) and another one, Griffithsin, is in clinical trials (against HIV). Actually, the pipeline from the identification of a compound with a certain bioactivity till the production of an approved drug involves pre-clinical tests, clinical trials in humans, and approval by FDA. This pipeline generally last 10–15 years, costing millions of dollars [254] and with less than 12% of the potential drugs receiving final approval [255] (Figure 7).

Recent COVID-19 pandemic event demonstrated the need of further necessity to invest in the search of new marine natural products with antiviral activity [256], as well as to explore the immense marine environment because several organisms are still underexplored. In view of using eco-friendly and eco-sustainable approaches to drug discovery (in line with the European perspective of a Blue Economy based on marine resources), microorganisms, especially those easily cultivable, have been considered emerging and promising sources of novel bioactives. In fact, isolation from macrorganisms should require massive collection practices. To overcome this problem, various approaches have been considered, from chemical synthesis to heterologous expression and production [257]. In addition, recent efforts have been also focused to discover new culturing methods to growth those that are considered “uncultivable organisms” and to isolate new [5] compounds from them. The ocean represents a huge untapped source of marine natural products with antiviral and other possible bioactivities useful for environment and human health.

## Figures and Tables

**Table 1:** The table report antiviral compounds/extracts from marine bacteria. CHIKV, Chikungunya virus; FNV, Fish Nodavirus; HSV-1, Herpes Simplex virus 1; WEEV, Western Equine Encephalitis virus; EPS, exopolysaccharides.

Compound/Extract	Organism	Which Virus?	Mechanism of Action	Reference
Butenolides	*Streptomyces* sp.	Anti-adenoviral	Undetermined	[12]
Debromoaplysiatoxin; 3methoxydebromoaplysiatoxin	*Trichodesmium erythraeum*	CHIKV	Target replication cycle after viral entry	[13]
Furan-2-yl acetate	*Streptomyces* VITSDK1 spp.	FNV	Undetermined	[14]
EPS	*Pseudoalteromonas* sp. *AM*	HSV-1	Undetermined	[15]
Chlorinated compounds	*Leptolyngbya*	Influenza A and B viruses	Inhibition neuraminidase activity and replication.	[16]
Antimycin A1a	*Streptomyces kaviengensis*	WEEV	inhibition of cellular mitochondrial electron transport chain	[17]

**Table 2:** The table report antiviral compounds/extracts from marine fungi. EV-71, Enterovirus 71; HIV, Human Immunodeficiency virus; HSV-1, Herpes Simplex virus 1; PRRSV, Porcine Reproductive and Respiratory virus; TMV, Tobacco Mosaic virus.

Compound/Extract	Organism	Which Virus?	Mechanism of Action	Reference
Grisephenone A	*Stachybotrys* sp.	EV-71	Not specified	[18]
Norlichexanthone-3,6,8-Trihydroxy-1-methylxanthone	*Stachybotrys* sp.	EV-71	Not specified	[18]
Stachybogrisephenone B	*Stachybotrys* sp.	EV-71	Not specified	[18]
Stachybotrins D	*Stachybotrys chartarum* MXH-X73	HIV-1	Not specified	[19,20]
Arisugacin A	*Aspergillus terreus* SCSGAF0162	HSV-1	Not specified	[21]
Aspergillipeptides D–E	*Aspergillus* sp. SCSIO 41501	HSV-1	Not specified	[22]
Aspernolide A	*Aspergillus terreus* SCSGAF0162	HSV-1	Not specified	[21]
Balticolid	*Ascomycetous* strain 222	HSV-1	Not specified	[23]
11a-dehydroxyisoterreulactone A	*Aspergillus terreus* SCSGAF0162	HSV-1	Not specified	[21]
Isobutyrolactone II	*Aspergillus terreus* SCSGAF0162	HSV-1	Not specified	[21]
Simplicilliumtide J	*Simplicillium obclavatum* EIODSF 020	HSV-1	Not specified	[24]
Verlamelins A-B	*Simplicillium obclavatum* EIODSF 020	HSV-1	Not specified	[24]
Asperterrestide A	*Aspergillus terreus* SCSGAF0162	Influenza A (H1N1 and H3N2) virus	Not specified	[25]
Cladosin C	*Cladosporium sphaerospermum* 2005-01-E3	Influenza A (H1N1) virus	Not specified	[26]
Cordyol C	*Aspergillus sydowii ZSDS1-F6*	Influenza A (H3N2) virus	Not specified	[27]
Diorcinol	*Aspergillus sydowii ZSDS1-F6*	Influenza A (H3N2) virus	Not specified	[27]
(*Z*)-5-(Hydroxymenthyl)-2-(6′)-methylhept-2′-en-2′-yl)-phenol	*Aspergillus sydowii ZSDS1-F6*	Influenza A (H3N2) virus	Not specified	[27]
Rubrolide S	*Aspergillus terreus* OUCMDZ-1925	Influenza A (H1N1) virus	Not specified	[28]
Sorbicatechols A and B	*Penicillium chrysogenum* PJX-17	Influenza A (H1N1) virus	Not specified	[29]
Alterporriol Q	*Alternaria* sp. ZJ-2008003	PRRSV	Not specified	[30]
Tetrahydroaltersolanol C	*Alternaria* sp. ZJ-2008003	PRRSV	Not specified	[30]
AGI-B4	*Neosartorya fischeri* 1008F1	TMV	Not specified	[31]
3,4-dihydroxybenzoic acid	*Neosartorya fischeri* 1008F1	TMV	Not specified	[31]
2-(4-hydroxybenzyl) quinazolin-4(3H)-one	*Penicillium oxalicum* 0312F1	TMV	Not specified	[32]
Methyl 4-hydroxyphenylacetate	*Penicillium oxalicum* 0312F1	TMV	Not specified	[32]
Penipanoid C – 2-(4-hydroxybenzoyl) quinazolin-4(3H)-one	*Penicillium oxalicum* 0312F1	TMV	Not specified	[32]

**Table 3:** Antiviral compounds from microalgae. HSV, Herpes Simplex virus; VSV, *Vescicular stomatitis* virus; EPS, exopolysaccharides; MGDG, monogalactosyl diacylglyceride.

Compound/Extract	Organism	Which Virus?	Mechanism of Action	Reference
EPS	*Porphyridium cruentum*	HSV, VSV and *Vaccinia virus*	Reduction of virus-induced cytopathogenicity	[73]
MGDG	*Coccomyxa* sp. KJ	HSV	Structural changes in virus particles	[74]
Marennine-like pigment	*Haslea karadagensis*	HSV	Inhibition of plaque formation	[75,76]
Polysaccharide-rich fraction	*Dunaliella* *Salina*	HSV	Inhibition of plaque formation	[62]
Sulfated polysaccharide p-KG03	*Gyrodinium impudium*	Influenza A virus (H1N1) and (H3N2)	Targeting virus particle attachment to cell surface receptors and internalization via virus–cell fusion	[77]

**Table 4:** Compounds with antiviral activity isolated from seaweeds. AIV, Avian Influenza virus; HSV-1, Herpes Simplex virus-1; ISA, Infectious Salmon Anemia; NDV, Newcastle disease virus; SARS-CoV, severe acute respiratory syndrome coronavirus replication; WSSV, White Spot Syndrome virus.

Compound/Extract	Organism	Which Virus?	Mechanism of Action	Reference
Sulfate polysaccharides	*Grateloupia filicina*	AIV	Targeting virus particle attachment to cell	[81]
Sulfate polysaccharides	*Ulva pertusa*	AIV	Targeting virus particle attachment to cell	[81]
Sulfate polysaccharides	*Sargassum qingdaoense*	AIV	Targeting virus particle attachment to cell	[81]
Sulfated glucuronorhamnan	*Monostroma nitidum*	Enteroviruses	Targeting virus particle attachment to cell	[82]
Alginates and fucoidan	*Sargassum naozhouense*	HSV-1	Targeting virus particle attachment to cell	[83]
Phlorofucofuroeckol	*Ulva clathrata*	Influenza A virus, H1N1, H3N2 and H9N2	neuraminidases activity inhibition	[84]
EPA, fatty acids, omega w-3	*Gracilaria chilensis*	ISA	Inhibition of viral replication	[85]
Ulvan	*Ulva clathrata*	NDV	Inhibited cell–cell fusion via a direct effect on the F0 protein	[86]
Fucoidan	*Cladosiphon okamuranus*	NDV	Inhibited cell–cell fusion	[86]
Diekol	*Ulva clathrata*	SARS-CoV	Inhibition of SARS-CoV 3CL^pro^	[87]
Ulvan	*Ulva* sp and *Eteromorpha* sp.	WSSV	Not reported	[88]

**Table 5:** The table report antiviral compounds/extracts from marine plants. HCV, Hepatitis C virus; HIV, Human Immunodeficiency virus; HSV-1, Herpes Simplex virus 1; TBE, Tick-Borne Encephalitis virus.

Compound/Extract	Organism	Which Virus?	Mechanism of Action	Reference
Thalassiolin D—diosmetin 7-*O*-β-glucoside-2″-sulphate	Seagrass—*Thalassia hemprichii*	HCV	Inhibition of HCV NS3-NS4A protease	[126]
Dimeric Alkylresorcinols	Mangrove—*Sonneratia hainanensis*	HIV-1	HIV-1 integrase inhibition	[127]
Asebotin, quercetin-3-*O*-β-d-xylopyranoside *trans*-caffeic acid	Seagrass—*Thalassodendron ciliatum*	HSV-1	Inhibition of plaque formation	[125]
Khayanolides	Mangrove—*Xylocarpus moluccensis*	Influenza A virus (H1N1)	Cytopathic effect inhibition	[128]
6′-*O*-rhamnosyl-(1‴→6″)-glucopyranosyl asebogenin—Thalassodendrone	Seagrass—*Thalassodendron ciliatum*	Influenza A virus	Reduce virus toxicity	[124]
Triterpenoids	Mangrove—*Sonneratia paracaseolaris*	Influenza A virus (H1N1)	Cytopathic effect inhibition	[129]
polyphenol complex	Seagrass—Zosteraceae	TBE virus	Reduction of virus titer	[130]

**Table 6:** The table report antiviral compounds/extracts from marine sponges. HBV, Hepatitis B virus; HCV, Hepatitis C virus; HIV-1, Human Immunodeficiency virus type 1; HSV-1, Herpes Simplex virus 1; SINV, Sindbis virus; TMV, Tobacco Mosaic virus.

Compound/Extract	Species	Which Virus?	Mechanism of Action	Reference
Metachromin A	*Dactylospongia metachromia*	HBV	Inhibition of HBV core promoter activity	[149]
Polybrominated diphenyl ethers	*Dysidea* sp.	HBV	Inhibition of HBV core promoter activity	[150]
Manoalide	*Luffariella variabilis*	HCV	Binding to a conserved helicase motif of the NS3 viral protein	[151]
Psammaplin A	*Psammaplysilla* sp., *Poecillastra* sp. and *Jaspis* sp.	HCV	Block of viral NS3 RNA helicase and ATPase activities	[152]
Aaptamine alkaloids	*Aaptos aaptos*	HIV-1	Not specified	[153]
Baculiferins C, E–H and K–N	*Iotrochota baculifera*	HIV-1	Interaction with Vif, APOBEC3G and gp41 proteins	[154]
Bengamide A	*Jaspis* cf. *coriacea*	HIV-1	Interaction with LTR NF-κB response elements	[155]
Mirabamides E-H	*Stelletta clavosa*	HIV-1	Not specified	[156]
Stellettapeptins A-B	*Stelletta* sp.	HIV-1	Not specified	[157]
Manzamine A	*Haliclona* and *Acanthostrongylophora* genera	HSV-1	Not specified	[158]
TSH fraction, halistanol sulfate and halistanol sulfate C	*Petromica citrina*	HSV-1	Inhibition of viral attachment/penetration and reduction of ICP27 and gD levels	[159]
Pateamine A	*Mycale* sp.	SINV	Block of viral mRNA translation by targeting eIF4A complex	[160]
Nortopsentins	*Spongosorites ruetzleri*	TMV	Not specified	[161]

**Table 7:** The table report antiviral compounds/extracts from mollusks. EBV, Epstein-Barr virus; HIV-1, Human Immunodeficiency virus type 1; HSV-1, Herpes Simplex virus 1; HSV-2, Herpes Simplex virus 2; OsHV-1, Ostreid herpesvirus 1; VHSV, Viral Hemorrhagic Septicemia virus.

Compound/Extrasct	Species	Which Virus?	Mechanism of Action	Reference
RvH and the functional units RvH1-a/RvH2-e	*Rapana venosa*	EBV	Not specified	[162,163]
Three hemocyanin fractions	*Haliotis rubra*	HIV-1	Binding to the viral surface through gD, gB, and gC glycoproteins	[164]
Cavortins	*Crassotea gigas*	HSV-1	Not specified	[165]
Hemolymph	*Haliotis laevigata*	HSV-1	Not specified	[166]
Myticin C, modified and nanoencapsulated	*Mytilus galloprovincialis*	OsHV-1, HSV-1/HSV-2	Not specified	[167]
Hemolymph and Myticin C	*Mytilus galloprovincialis*	VHSV	Not specified	[168]

**Table 8:** The table report antiviral compounds/extracts from cnidarians, CHIKV, Chikungunya virus; HCMV, Human Cytomegalovirus; HSV-1, Herpes Simplex virus 1; RSV, Respiratory Syncytial virus.

Compound/Extract	Species	Which Virus?	Mechanism of Action	Reference
Norcembranoids and sesquiterpenoid	*Sinularia kavarattiensis*	CHIKV	Not specified	[169]
Briacavatolides C-F	*Briareum excavatum*	HCMV	Not specified	[170,171]
Durumolide J	*Lobophytum durum*	HCMV	Not specified	[172]
Ehrenbergol C and acetyl ehrenberoxide B	*Sarcophyton ehrenbergi*	HCMV	Not specified	[173]
Gyrosanols A and B	*Sinularia gyrosa*	HCMV	Not specified	[174]
Hipposterone N	*Isis hippuris*	HCMV	Not specified	[175]
Secocembranoid	*Lobophytum crassum*	HCMV	Not specified	[176]
Zoanthoxanthins	*Echinogorgia pseudossapo*	HSV-1	Not specified	[177]
Polyhydroxylated sterol and ceramide derivatives	*Sinularia candidula*	Influenza A virus (H5N1)	Not specified	[178]
Polyhydroxylated steroids	*Sarcophyton* sp.	Influenza A virus (H1N1)	Not specified	[179]
Echrebsteroids A–C	*Echinogorgia rebekka*	RSV	Not specified	[180]

**Table 9:** The table report antiviral compounds/extracts from crustaceans. FCV-F9, Feline Calicivirus F9; WSSV, White Spot Syndrome virus; VP28, viral envelope protein.

Compound/Extract	Species	Which Virus?	Mechanism of Action	Reference
Chitosan	Several crustacean species	MS2/phi X174 phages and FCV-F9	Not specified	[181]
Crustin, Sp-Crus6	*Scylla paramamosain*	WSSV	Not specified	[182]
Hemocyanin, LvHcL48	*Litopenaeus vannamei*	WSSV	Interaction to the viral envelope protein VP28	[183]
Hemocyte proteins, Sp-ALFs	*Scylla paramamosain*	WSSV	Not specified	[184]
Peroxinectin analog, Sp-PX	*Scylla paramamosain*	WSSV	Not specified	[185]
Scygonadin	*Scylla paramamosain*	WSSV	Not specified	[186]
SWD, LvSWD3	*Litopenaeus vannamei*	WSSV	Not specified	[187]
β-thymosin-repeat proteins	*Marsupenaeus japonicus*	WSSV	Not specified	[188]

**Table 10:** The table report antiviral compounds/extracts from echinoderms. HBV, Hepatitis B virus; HIV-1, Human immunodeficiency virus type 1; HSV-1, Herpes simplex virus 1; HSV-2, Herpes simplex virus 2, PrV, Pseudorabies virus; PLA2, phospholipase A2.

Compound/Extract	Species	Which Virus?	Mechanism of Action	Reference
Acidic mucopolysaccharide, SJAMP	*Stichopus japonicus*	HBV	Not specified	[189]
AP-PLA2 from crude venom	*Acanthaster planci*	HIV-1	Not specified	[190]
Comaparvin	*Capillaster multiradiatus*	HIV-1	Not specified	[191]
Seven hydrolysates	*Cucumaria frondosa*	HSV-1	Not specified	[192]
Sulfated sterols	Echinoderms from cold waters	HSV-1, HSV-2 and PrV	Not specified	[193]

**Table 11:** The table report antiviral compounds/extracts from tunicates. HIV-1, Human Immunodeficiency virus 1; HSV-1, Herpes simplex virus 1; JEV, Japanese Encephalitis virus; IPV, Inactivated Polio vaccine; TMV, Tobacco Mosaic virus.

Compound/Extract	Species	Which Virus?	Mechanism of Action	Reference
Prunolide A and Cadiolide B	*Synoicum prunum* and *Botryllus* sp.	JEV	Not specified	[194]
Mollamide F, Molleurea A and Mollamide E	*Didemnum molle*	HIV-1	Inhibition of viral replication and HIV-1 integrase	[195]
Eudistomin C	*Ritterella sigillinoides*	HSV-1 and IPV-1	Interaction to the uS11-containing small ribosomal subunit	[196]
Polycarpaurines A and C	*Polycarpa aurata*	TMV	Not specified	[197]
